# PATTERNS OF PSYCHOACTIVE MEDICATION USE IN COMMUNITY-DWELLING OLDER ADULTS IN THE UNITED STATES IN 2015

**DOI:** 10.1093/geroni/igz038.2608

**Published:** 2019-11-08

**Authors:** Rashmita Bajracharya, Danya Qato

**Affiliations:** 1 University of Maryland, Baltimore, Maryland, United States; 2 University of Maryland Baltimore, School of Medicine, Maryland, United States

## Abstract

Per the 2015 Beer’s Criteria, most psychoactive medications are identified as potentially inappropriate for use in older adults as this population is especially vulnerable to the potential adverse effects associated with psychoactive medications, including sedation, anticholinergic effects, and falls. Past studies found increasing use of psychoactive medications in community-dwelling older adults; however, patterns of use by other sociodemographic, socioeconomic, and clinical subgroups have not been explored. This is a cross-sectional analysis of 2015 Medical Expenditure Panel Survey in a sample of 6122 older adults (60-85 years). We utilized Andersen’s Behavioral Model of Health Services Utilization to guide logistic regression model development and estimated odds ratios (OR) with 95% confidence intervals (CI) to quantify the association between psychoactive use and predisposing(sex and race); enabling(marital status, education, poverty, insurance); and need-based[multi-morbidity and activities of daily living (ADL) limitations] factors. Over 30% of older adults in the U.S. reported taking a psychoactive medication in 2015. Prevalence of use was significantly higher in women (35.9%), the unmarried(34.1%), low-income(35.7%), white(34.0%), multimorbid (32.0%), and ADL limitation groups (45.9%) compared to men, married, high-income, other races, not multimorbid, and no ADL limitations groups, respectively. Female sex [OR=1.62(1.38-1.91)], low-income [OR=1.30(1.04-1.6)], multimorbidity [OR=3.2(2.6-3.9)], and ADL limitations [OR=2.2(1.7-2.8)] were identified as independent predictors of psychoactive use. There is differential use of psychoactive medications by sociodemographic, socioeconomic, and clinical factors. Given the increased complexity of pharmacotherapy regimens, especially in those with multimorbidity and ADL limitations, improved efforts aimed at prudent use of psychoactive medications should be intensified.

